# Partial Derivative Approach to the Integral Transform for the Function Space in the Banach Algebra

**DOI:** 10.3390/e22091047

**Published:** 2020-09-18

**Authors:** Kim Young Sik

**Affiliations:** Department of Mathematics, Hanyang University, Seoul 04763, Korea; yoskim@hanyang.ac.kr

**Keywords:** function space, integral transform, 28 C 20

## Abstract

We investigate some relationships among the integral transform, the function space integral and the first variation of the partial derivative approach in the Banach algebra defined on the function space. We prove that the function space integral and the integral transform of the partial derivative in some Banach algebra can be expanded as the limit of a sequence of function space integrals.

## 1. Introduction

The first variation defined by the partial derivative approach was defined in [1]. Relationships among the Function space integral and transformations and translations were developed in [2,3,4]. Integral transforms for the function space were expanded upon in [5,6,7,8,9].

A change of scale formula and a scale factor for the Wiener integral were expanded in [10,11,12] and in [13] and in [14].

Relationships among the function space integral and the integral transform and the first variation were expanded in [13,15,16] and in [17,18]

In this paper, we expand those relationships among the function space integral, the integral transform and the first variation into the Banach algebra [19].

## 2. Preliminaries

Let $C_{0}\left[0,T\right]$ be the class of real-valued continuous functions *x* on [0,T] with x(0)=0, which is a function space. Let *M* denote the class of all Wiener measurable subsets of $C_{0}\left[0,T\right]$ and let *m* denote the Wiener measure. Then $\left(C_{0}\left[0,T\right],M,m\right)$ is a complete measure space and

$$E_{x}\left[F\left(x\right)\right]=\int_{C_{0}\left[0,T\right]}\;F\left(x\right)\;\;dm\left(x\right)\;$$

is called **the Wiener integral** of a function *F* defined on the function space $C_{0}\left[0,T\right]$.

A subset *E* of $C_{0}\left[0,T\right]$ is said to be scale-invariant measurable provided ρE∈M for all ρ>0 and a scale invariant measurable set *N* is said to be scale-invariant null provided m(ρN)=0 for each ρ>0. A property that holds except on a scale-invariant null set is said to hold scale-invariant almost everywhere (s-a.e.). If two functions *F* and *G* are equal s-a.e., we write F≈G.

**Definition** **1.** *For the definition of the analytic Wiener integral and the analytic Feynman integral, see Definition 1 in [18]: $C_{+}=\left\{\lambda |Re\left(\lambda \right)>0\right\}$ and $C_{+}^{∼}=\left\{\lambda |Re\left(\lambda \right)\geq 0\right\}$. For real λ>0,*

(1) $$J_{F}\left(\lambda \right)=E_{x}(F\left(\lambda^{-\frac{1}{2}}x\right)).$$

*For each $z\in C_{+}$, the analytic Wiener integral is defined by*

(2) $$E_{x}^{anw_{z}}\left[F\left(x\right)\right]=E_{x}(F\left(z^{-\frac{1}{2}}x\right))=J_{F}^{*}\left(z\right).$$

*Whenever z→−iq through $C_{+}$, the analytic Feynman integral is defined by*

(3) $$E_{x}^{anf_{q}}\left[F\left(x\right)\right]=\lim_{z\to -iq}E_{x}^{anw_{z}}\left[F\left(x\right)\right],$$

*where $i^{2}=-1$.*

**Notation** **1.** *For $\lambda \in C_{+}$ and for $s-a.e.y\in C_{0}\left[0,T\right]$, let*

(4) $$\left(\;T_{\lambda }\left(F\right)\;\right)\left(y\right)=E_{x}^{anw_{\lambda }}\left[F\left(\;x+y\;\right)\right].$$

**Definition** **2.** *For the $L_{1}$-analytic Fourier–Feynmann transform, see Definition 2 in [5]:*

(5) $$\left(\;T_{q}^{\left(1\right)}\left(F\right)\;\right)\left(y\right)=\lim_{\lambda \to -iq}E_{x}^{anw_{\lambda }}\left[F\left(\;x+y\;\right)\right]=E_{x}^{anf_{q}}\left[F\left(\;x+y\;\right)\right]\;,$$

*whenever λ→−iq through $C_{+}$ (if it exists). See [5,9].*

**Definition** **3** (Ref. [1]). *The first variation of a Wiener measurable functional F in the direction $w\in C_{0}\left[0,T\right]$ which is defined by the partial derivative as*

(6) $$\delta F\left(x|w\right)=\frac{\partial }{\partial h}{F\left(x+hw\right)|}_{h=0}\;.$$

*We will denote it by [D,F,x,w].*

**Remark** **1.** *For $a\in C_{+}$ and b∈R,*

(7) $$\begin{array}{ccc} & & \int_{R}\exp\left\{-au^{2}+ibu\right\}\;du=\sqrt{\frac{\pi }{a}}\exp\left\{-\frac{b^{2}}{4a}\right\}\;. \end{array}$$

## 3. Results (1). On $C_{0}\left[0,T\right]$

Let

(8) $$\begin{array}{ccc} & & F\left(x\right)=\int_{L_{2}\left[0,T\right]}\;\exp\{i\;\left[\;I,v\left(t\right),x\left(t\right)\;\right]\}\;df\left(v\right) \end{array}$$

in some Banach algebra S defined on $C_{0}\left[0,T\right]$ in [19], where $\left[I,v\left(t\right),x\left(t\right)\right]=\int_{0}^{T}v\left(t\right)dx\left(t\right)$ and assume that $\int_{L_{2}\left[0,T\right]}{\left||v|\right|}_{2}\;d\left|f\right|\left(v\right)\;<\;\infty$.

Suppose that formulas in this section hold for $s-a.e.w\in C_{0}\left[0,T\right]$ and for $s-a.e.y\in C_{0}\left[0,T\right]$.

**Lemma** **1.** (9) $$\begin{array}{ccc} & & \left[D,F,x,w\right]=\int_{L_{2}\left[0,T\right]}\;(i\;\left[I,v\left(t\right),w\left(t\right)\right])\exp\{i\;\left[I,v\left(t\right),x\left(t\right)\right]\}df\left(v\right),\; \end{array}$$

*where $\left[I,v\left(t\right),w\left(t\right)\right]=\int_{0}^{T}v\left(t\right)dw\left(t\right).$*

**Proof** **.** **By Equation (6).**

(10) $$\begin{array}{ccc} & & \left[D,F,x,w\right]\\ & = & \frac{\partial }{\partial h}{\;F\left(\;x+hw\;\right)|}_{h=0}\\ & = & \frac{\partial }{\partial h}\;\int_{L_{2}\left[0,T\right]}{\;\;\exp\{\;i\;h\;\left[I,v\left(t\right),w\left(t\right)\right]\;+i\;\left[I,v\left(t\right),x\left(t\right)\right]\}\;\;df\left(v\right)\;|}_{h=0}\\ & = & \int_{L_{2}\left[0,T\right]}\;(i\;\left[I,v\left(t\right),w\left(t\right)\right]\;)\exp\{\;i\;\left[I,v\left(t\right),x\left(t\right)\right]\}df\left(v\right)\;. \end{array}$$

and

(11) $$\begin{array}{ccc} & & \left|\;E_{w}(\int_{L_{2}\left[0,T\right]}\;\left[I,v\left(t\right),w\left(t\right)\right]\;\;\cdot \;\exp\{i\;\left[I,v\left(t\right),x\left(t\right)\right]\;\}\;df\left(v\right)\;)\;\right|\\ & \leq & E_{w}(\int_{L_{2}\left[0,T\right]}\;|\;\left[I,v\left(t\right),w\left(t\right)\right]\;|\;d\left|f\right|\left(v\right)). \end{array}$$

Then

(12) $$\begin{array}{ccc} & & E_{w}(\int_{L_{2}\left[0,T\right]}\;|\;\left[I,v\left(t\right),w\left(t\right)\right]\;|\;d\left|f\right|\left(v\right))\\ & = & \int_{L_{2}\left[0,T\right]}\;E_{w}(|\;\left[I,v\left(t\right),w\left(t\right)\right]\;|)\;d\left|f\right|\left(v\right)\\ & = & \int_{L_{2}\left[0,T\right]}[\frac{1}{\sqrt{{2\pi \;|v||}_{2}^{2}}}\int_{-\infty }^{+\infty }\;|u|\;\exp\{-\frac{u^{2}}{{2||v||}_{2}^{2}}\}\;du\;]\;d\left|f\right|\left(v\right)\\ & = & \sqrt{\frac{2}{\pi }}\;\int_{L_{2}\left[0,T\right]}{\;||v||}_{2}\;d\left|f\right|\left(v\right)\;<\;\infty \; \end{array}$$

where $E_{w}(F\left(w\right))=\int_{C_{0}\left[0,T\right]}\;F\left(w\right)\;dm\left(w\right).$ So,

$$\int_{L_{2}\left[0,T\right]}\;|\;\left[I,v\left(t\right),w\left(t\right)\right]\;|\;d\left|f\right|\left(v\right)\;<\;\infty .$$

Therefore, [D,F,x,w] exists.  ☐

**Theorem** **1.** (13) $$\begin{array}{ccc} & [1]. & E_{x}^{anw_{z}}(\left[D,F,x+y,w\right])\\ & & =\int_{L_{2}\left[0,T\right]}\;(i\;\left[I,v\left(t\right),w\left(t\right)\right])\exp\{-\frac{1}{2z}\int_{0}^{T}v^{2}\left(s\right)ds+i\;\left[I,v\left(t\right),y\left(t\right)\right]\;\}df\left(v\right)\; \end{array}$$

(14) $$\begin{array}{ccc} & [2]. & E_{x}^{anf_{q}}(\;\left[D,F,x+y,w\right]\;)\;\\ & & =\int_{L_{2}\left[0,T\right]}\;(i\;\left[I,v\left(t\right),w\left(t\right)\right]\;)\exp\{-\frac{i}{2q}\int_{0}^{T}\;v^{2}\left(s\right)ds+i\;\left[I,v\left(t\right),y\left(t\right)\right]\;\}df\left(v\right)\;, \end{array}$$

*where [D,F,x+y,w]=δF(x+y|w).*

**Proof.**  [1]. For $z\;\in \;C_{+}$,

(15) $$\begin{array}{ccc} & & E_{x}^{anw_{z}}(\left[D,F,x+y,w\right])\\ & = & E_{x}\;(\left[D,F,z^{-\frac{1}{2}}x\;,y,w\;\right])\\ & = & E_{x}(\int_{L_{2}\left[0,T\right]}\;(i\;\left[I,v\left(t\right),w\left(t\right)\right]\;)\;\\ & & \;\;\;\cdot \;\exp\{iz^{-\frac{1}{2}}\left[I,v\left(t\right),x\left(t\right)\right]\;+\;i\;\left[I,v\left(t\right),y\left(t\right)\right]\}\;df\left(v\right))\\ & = & \int_{L_{2}\left[0,T\right]}\;(i\;\left[I,v\left(t\right),w\left(t\right)\right])\\ & & \;\;\;\cdot E_{x}(\exp\{iz^{-\frac{1}{2}}\left[I,v\left(t\right),x\left(t\right)\right]\})\cdot \exp\{i\;\left[I,v\left(t\right),y\left(t\right)\right]\}\;df\left(v\right)\\ & = & \int_{L_{2}\left[0,T\right]}(i\;\left[I,v\left(t\right),w\left(t\right)\right]\;)\cdot \;\exp\{-\frac{1}{2z}{\;||v||}_{2}^{2}\;+\;i\;\left[I,v\left(t\right),y\left(t\right)\right]\;\}df\left(v\right)\;. \end{array}$$

[2].

(16) $$\begin{array}{ccc} & & E_{x}^{anf_{q}}(\left[D,F,x+y,w\right])\\ & = & \lim_{z\to -iq}\;E_{x}^{anw_{z}}(\left[D,F,x+y,w\;\right])\\ & = & \lim_{z\to -iq}\;\int_{L_{2}\left[0,T\right]}\;(i\;\left[I,v\left(t\right),w\left(t\right)\right]\;)\;\cdot \;\exp\{(-\frac{1}{2z}{\;||v||}_{2}^{2}\;+\;i\;\left[I,v\left(t\right),y\left(t\right)\right]\;\}df\left(v\right)\\ & = & \int_{L_{2}\left[0,T\right]}\;(\;i\;\left[I,v\left(t\right),w\left(t\right)\right]\;)\;\cdot \;\exp\{-\frac{i}{2q}\left[\int_{0}^{T}\;v^{2}\left(s\right)ds\right]+i\left[I,v\left(t\right),y\left(t\right)\right]\;\}df\left(v\right)\;\;. \end{array}$$

 ☐

**Lemma** **2.** *For $\lambda \in C_{+}$,*

(17) $$\begin{array}{ccc} & & \exp\{\frac{1-\lambda }{2}\;\sum_{k=1}^{n}\;{\left[I,\phi_{k}\left(t\right),x\left(t\right)\right]}^{2}\;\}\;\cdot \;\left[D,F,x+y,w\right] \end{array}$$

*is a Wiener integrable function of $x\in C_{0}\left[0,T\right]$.*

**Proof.**  (18) $$\begin{array}{ccc} & & E_{x}(\exp\{\frac{1-\lambda }{2}\sum_{k=1}^{n}\;{\left[I,\phi_{k}\left(t\right),x\left(t\right)\right]}^{2}\}\;\left[D,F,x+y,w\right])\\ & = & E_{x}(\exp\{\frac{1-\lambda }{2}\sum_{k=1}^{n}\;{\left[I,\phi_{k}\left(t\right),x\left(t\right)\right]}^{2}\}\\ & & \;\cdot \;\;[\;\int_{L_{2}\left[0,T\right]}\;(i\;\left[I,v\left(t\right),w\left(t\right)\right]\;)\cdot \;\exp\{i\;\left[I,v\left(t\right),x\left(t\right)\right]+\;i\;\left[I,v\left(t\right),y\left(t\right)\right]\}\;df\left(v\right)])\\ & = & \int_{L_{2}\left[0,T\right]}\;(i\;\left[I,v\left(t\right),w\left(t\right)\right]\;)\;\cdot \;E_{x}(\exp\{\frac{1-\lambda }{2}\sum_{k=1}^{n}\;\;{\left[I,\phi_{k}\left(t\right),x\left(t\right)\right]}^{2}\;\}\\ & & \;\;\cdot \exp\{i\;[I,v(t),x(t)]\;+\;i\;[I,v(t),y(t)]\;\})\;df(v)\;. \end{array}$$

and

(19) $$\begin{array}{ccc} & & E_{x}(\exp\{\frac{1-\lambda }{2}\sum_{k=1}^{n}\;{\left[I,\phi_{k}\left(t\right),x\left(t\right)\right]}^{2}\;\}\cdot \exp\{i\;\left[I,v\left(t\right),x\left(t\right)\right]+\;i\;\left[I,v\left(t\right),y\left(t\right)\right]\})\\ & \leq & E_{x}(\exp\{\frac{1-\lambda }{2}\sum_{k=1}^{n}\;{\left[I,\phi_{k}\left(t\right),x\left(t\right)\right]}^{2}\})\\ & = & {\left(2\pi \right)}^{-\frac{n}{2}}\int_{R^{n}}\;\exp\{-\frac{\lambda }{2}\sum_{k=1}^{n}\;u_{k}^{2}\}\;d\vec{u}\\ & = & {\left(2\pi \right)}^{-\frac{n}{2}}\;\cdot \;{\left(\frac{2\pi }{\lambda }\right)}^{\frac{n}{2}}\\ & = & \lambda^{-\frac{n}{2}}\;. \end{array}$$

Therefore,

(20) $$\begin{array}{ccc} & & \left|\;\int_{L_{2}\left[0,T\right]}\;\;(\;i\;\left[I,v\left(t\right),w\left(t\right)\right]\;)\;\cdot \;E_{x}(\exp\{\frac{1-\lambda }{2}\sum_{k=1}^{n}\;{\left[I,\phi_{k}\left(t\right),x\left(t\right)\right]}^{2}\right\}\\ & & \;\;\cdot \exp\{i\;[I,v(t),x(t)]+\;i\;[I,v(t),y(t)]\}\;)\;df(v)\;|\\ & \leq & \lambda^{-\frac{n}{2}}\;\cdot \;\int_{L_{2}\left[0,T\right]}\;|\;\left[I,v\left(t\right),w\left(t\right)\right]\;|\;d\left|f\right|\left(v\right)\;. \end{array}$$

By the Wiener integration theorem,

(21) $$\begin{array}{ccc} & & E_{w}(\int_{L_{2}\left[0,T\right]}\;|\;\left[I,v\left(t\right),w\left(t\right)\right]\;|\;d\left|f\right|\left(v\right)\;)\\ & = & \int_{L_{2}\left[0,T\right]}\;\;[\;\;\frac{1}{\sqrt{{2\;\pi ||v||}_{2}^{2}}}\;\cdot \;\;\int_{-\infty }^{+\infty }\;\;|u|\;\cdot \;\exp\{-\frac{u^{2}}{{2||v||}_{2}^{2}}\;\}\;du\;\;]\;\;d\left|f\right|\left(v\right)\\ & = & {\left(\frac{2}{\pi }\right)}^{\frac{1}{2}}\;\int_{L_{2}\left[0,T\right]}{\;||v||}_{2}\;\;d\left|f\right|\left(v\right)<\;\infty \;\;. \end{array}$$

 ☐

**Lemma** **3** (Ref. [12]). *Let ${\left\{\phi_{j}\right\}}_{j=1}^{m}$ be an orthonormal set in $L_{2}\left[0,T\right]$. Then for $v\in L_{2}\left[0,T\right]$ and for $\lambda \in C_{+}$,*

(22) $$\begin{array}{ccc} & & E_{x}(\exp\{\frac{1-\lambda }{2}\sum_{k=1}^{m}\;{\left[I,\phi_{k}\left(t\right),x\left(t\right)\right]}^{2}+i\;\left[I,v\left(t\right),x\left(t\right)\right]\})\\ & = & \lambda^{-\frac{m}{2}}\;\cdot \;\exp\{-\frac{1-\lambda }{2\lambda }\sum_{k=1}^{m}{\left(\;\int_{0}^{T}\phi_{k}\left(s\right)v\left(s\right)ds\;\right)}^{2}-\frac{1}{2}\;\int_{0}^{T}v^{2}\left(s\right)ds\;]\}\;. \end{array}$$

**Theorem** **2.** *For $z\in C_{+}$,*

(23) $$\begin{array}{ccc} & & E_{x}^{anw_{z}}(\left[D,F,x+y,w\right])\\ & = & \lim_{n\to \infty }z^{\frac{n}{2}}\;\cdot \;E_{x}(\exp\{\frac{z-1}{2\;z}\sum_{k=1}^{n}\;{\left[I,\;\phi_{k}\left(t\right)\;,x\left(t\right)\;\right]}^{2}\}\;\left[D,F,x+y,w\right]) \end{array}$$

**Proof** **by Lemma 1.** (24) $$\begin{array}{ccc} & & \left[D,F,z^{-\frac{1}{2}}x+y,w\right)\\ & = & \int_{L_{2}\left[0,T\right]}\;(i\;\left[I,v\left(t\right),w\left(t\right)\right])\;\exp\{\;i\;z^{-\frac{1}{2}}\left[I,v\left(t\right),x\left(t\right)\right]\;+i\;\left[I,v\left(t\right),y\left(t\right)\right]\;\}df\left(v\right)\;. \end{array}$$

By Lemma 3,

(25) $$\begin{array}{ccc} & & \lim_{n\to \infty }\;z^{\frac{n}{2}}\;\cdot E_{x}(\exp\{\frac{z-1}{2\;z}\sum_{k=1}^{n}{\left[I,\;\phi_{k}\left(t\right),\;x\left(t\right)\;\right]}^{2}\}\;\left[D,F,x+y,w\right])\\ & = & \lim_{n\to \infty }z^{\frac{n}{2}}\;\int_{L_{2}\left[0,T\right]}\;E_{x}(\exp\{\frac{z-1}{2\;z}\sum_{k=1}^{n}{\left[I,\phi_{k}\left(t\right),x\left(t\right)\right]}^{2}\;+i\;\left[I,v\left(t\right),x\left(t\right)\right]\;\})\\ & & \;\;\;\;\;\cdot \;(\;i\;[I,v(t),w(t)]\;)\;\cdot \;\exp\{i\;[I,v(t),y(t)]\;\}df(v)\\ & = & \lim_{n\to \infty }z^{\frac{n}{2}}\;\int_{L_{2}\left[0,T\right]}\;z^{-\frac{n}{2}}\;\;\exp\{\frac{z-1}{2\;z}\sum_{k=1}^{m}\;\int_{0}^{T}\phi_{k}\left(s\right)\;v\left(s\right),ds\;{]}^{2}\;-\;\frac{1}{2}\int_{0}^{T}v^{2}\left(t\right)dt]\}\\ & & \;\;\;\;\;\cdot \;(\;i\;[I,v(t),w(t)]\;)\cdot \;\;\exp\{\;i\;[I,v(t),y(t)]\;\}df(v)\\ & = & \lim_{n\to \infty }\;\int_{L_{2}\left[0,T\right]}\;(\;i\;\left[\;I,v\left(t\right),w\left(t\right)\;\right]\;)\exp\{-\frac{1}{2\;z}\sum_{k=1}^{n}{\left[\int_{0}^{T}\;\phi_{k}\left(s\right)\;v\left(s\right)\;ds\;\right]}^{2}\;\}\;\\ & & \;\;\;\;\cdot \;\exp\{\;\frac{1}{2}\sum_{k=1}^{n}\;{\left[\;\int_{0}^{T}\;\phi_{k}\left(s\right)\;v\left(s\right)\;ds\;\right]}^{2}\;-\;\frac{1}{2}\int_{0}^{T}\;v^{2}\left(t\right)\;dt\}\\ & & \;\;\;\;\cdot \;\;\exp\{i\;[I,v(t),y(t)]\;\}df(v)\\ & = & \int_{L_{2}\left[0,T\right]}\;(i\;\left[\;I,\;v\left(t\right),w\left(t\right)\;\right]\;)\;\exp\{-\frac{1}{2z}{\;||v||}_{2}^{2}\;+\;\left[I,v\left(t\right),y\left(t\right)\right]\;\}\;df\left(v\right)\\ & = & \int_{L_{2}\left[0,T\right]}\;(\;i\;\left[I,v\left(t\right),w\left(t\right)\right]\;)\;\cdot \;E_{x}(\exp\{iz^{-\frac{1}{2}}\left[I,v\left(t\right),x\left(t\right)\right]\;\})\;\\ & & \;\;\;\;\;\cdot \;\;\exp\{i\;[I,v(t),y(t)]\;\}df(v)\\ & = & E_{x}(\int_{L_{2}\left[0,T\right]}\;\;(\;i\;\left[I,v\left(t\right),w\left(t\right)\right]\;)\\ & & \;\;\;\;\cdot \;\exp\{\;iz^{-\frac{1}{2}}\left[I,v\left(t\right),x\left(t\right)\right]\;+\;i\;\left[I,v\left(t\right),y\left(t\right)\right]\;\}df\left(v\right)\;)\\ & = & E_{x}(\left[D,F,z^{-\frac{1}{2}}x+y,w\right])\\ & = & E_{x}^{anw_{z}}(\left[D,F,x+y,w\right])\;.\;\; \end{array}$$

 ☐

**Theorem** **3.** *For real ρ>0,*

(26) $$\begin{array}{ccc} & & E_{x}\left(\;D\left[F,\rho \;x+y,w\right)]\right)\\ & = & \lim_{n\to \infty }\rho^{-n}\;\cdot \;E_{x}(\exp\{\frac{\rho^{2}-1}{2\rho^{2}}\sum_{k=1}^{m}{\left[\;I,\phi_{k}\left(t\right),x\left(t\right)\;\right]}^{2}\}\;D\left[F,x+y,w\right])\;. \end{array}$$

**Proof.**  For real λ>0,

(27) $$\begin{array}{ccc} & & E_{x}^{anw_{\lambda }}(\left[D,F,x+y,w\right])\\ & \equiv & E_{x}(\left[D,F,\lambda^{-\frac{1}{2}}x+y,w\right])\\ & = & \lim_{n\to \infty }\lambda^{\frac{n}{2}}\;\cdot \;E_{x}(\exp\{\frac{\lambda -1}{2\lambda }\sum_{k=1}^{m}\;{\left[I,\phi_{k}\left(t\right),x\left(t\right)\;\right]}^{2}\}\;D\left[\;F,x+y,w\;\right])\;. \end{array}$$

Taking $\lambda =\rho^{-2}$, we have the result.  ☐

**Theorem** **4.** (28) $$\begin{array}{ccc} & & E_{x}^{anf_{q}}\left(D\left[F,x+y,w\right]\;)\;\right)\\ & = & \lim_{n\to \infty }{\lambda_{n}}^{\frac{n}{2}}\;\cdot \;E_{x}(\exp\{\frac{\lambda_{n}-1}{2\lambda_{n}}\sum_{k=1}^{m}{\left[I,\phi_{k}\left(t\right),x\left(t\right)\right]}^{2}\;\}\;D\left[F,x+y,w\right])\;, \end{array}$$

*whenever $\{\lambda_{n}\}\;\to -\;iq$ through $C_{+}$.*

**Proof** **.** **by Theorem 2**,

(29) $$\begin{array}{ccc} & & E_{x}^{anf_{q}}\left(D\left[F,x+y,w\right]\;)\;\right)\\ & = & \lim_{n\to \infty }E_{x}^{anw_{\lambda_{n}}}(D\left[F,x+y,w\right])\\ & = & \lim_{n\to \infty }{\lambda_{n}}^{\frac{n}{2}}\;\cdot \;E_{x}(\exp\{\frac{\lambda_{n}-1}{2\lambda_{n}}\sum_{k=1}^{m}{\left[I,\phi_{k}\left(t\right),x\left(t\right)\right]}^{2}\;\}\;D\left[F,x+y,w\right])\;. \end{array}$$

 ☐

## 4. Results (2). on $C_{0}^{\nu }\left[0,T\right]$

In this section, we expand the result about the function:

(30) $$\begin{array}{ccc} & & F\left(\vec{x}\right)=\int_{L_{2}^{\nu }\left[0,T\right]}\;\exp\{i\;\sum_{j=1}^{\nu }\left[\;I,v_{j}\left(t\right),x_{j}\left(t\right)\;\right]\}\;df\left(\vec{v}\right) \end{array}$$

in some Banach algebra $S^{\prime }$ defined on $C_{0}^{\nu }\left[0,T\right]$ in [14].

Let $\vec{w}=\left(w_{1},\cdots ,w_{\nu }\right)$, where $w_{j}\in C_{0}\left[0,T\right]$ is absolutely continuous on [0,T] and $w_{j}^{{}^{\prime }}\left(t\right)\in L_{2}\left[0,T\right]$ for 1≤j≤ν. Suppose also that $M={Max}_{1\leq j\leq \nu }\left|\right|w_{j}^{{}^{\prime }}{\left|\right|}_{2}<\infty$ and we assume that $\int_{L_{2}^{\nu }\left[0,T\right]}\;\sum_{j=1}^{\nu }\left|\right|v_{j}{\left|\right|}_{2}\;\;d\left|f\right|\left(\vec{v}\right)\;<\;\infty$.

Suppose that formulas in this section hold $s-a.e.\vec{w}\in C_{0}^{\nu }\left[0,T\right]$ and for $s-a.\vec{y}\in C_{0}^{\nu }\left[0,T\right]$.

**Lemma** **4.** (31) $$\begin{array}{ccc} & & D\left[F,\vec{x},\vec{w}\right]\;=\int_{L_{2}^{\nu }\left[0,T\right]}\;(\;i\;\sum_{j=1}^{\nu }\left[\;I,v_{j}\left(t\right),w_{j}\left(t\right)\;\right]\;)\;\exp\{i\;\sum_{j=1}^{\nu }\;\left[\;I,v_{j}\left(t\right),x_{j}\left(t\right)\;\right]\}\;df\left(\vec{v}\right).\; \end{array}$$

**Proof** **.** **By Equation (6).**

(32) $$\begin{array}{ccc} & & D[F,\vec{x},\vec{w}]\\ & = & \frac{\partial }{\partial h}\;F\left(\;\vec{x}+h\vec{w}\;\right){|}_{h=0}\\ & = & \frac{\partial }{\partial h}\;\int_{L_{2}^{\nu }\left[0,T\right]}\;\;\exp\{\;i\;h\;\sum_{j=1}^{\nu }\;\left[\;I,v_{j}\left(t\right),w_{j}\left(t\right)\;\right]\;\;+i\;\sum_{j=1}^{\nu }\;\left[\;I,v_{j}\left(t\right),x_{j}\left(t\right)\;\right]\;\}\;\;df\left(\vec{v}\right){\;|}_{h=0}\\ & = & \int_{L_{2}^{\nu }\left[0,T\right]}\;(\;i\;\sum_{j=1}^{\nu }\;\left[\;I,v_{j}\left(t\right),w_{j}\left(t\right)\;\right]\;)\;\exp\{\;i\;\sum_{j=1}^{\nu }\;\left[\;I,v_{j}\left(t\right),x_{j}\left(t\right)\;\right]\;\}\;\;df\left(\vec{v}\right)\;. \end{array}$$

We know that the Paley–Wiener–Zygmund integral equals to the Riemann–Stieltzes integral

$$\int_{0}^{T}f\left(t\right)\;dg\left(t\right)\;=\;\int_{0}^{T}f\left(t\right)\;g^{\prime }\left(t\right)\;dt\;,$$

if *g* is absolutely continuous in [0,T] with $g^{\prime }\left(t\right)\in L_{2}\left[0,T\right]$.

For 1≤j≤ν, $\int_{0}^{T}\;v_{j}\left(t\right)dw_{j}\left(t\right)=\int_{0}^{T}\;v_{j}\left(t\right)w_{j}^{{}^{\prime }}\left(t\right)\;dt$. Therefore

(33) $$\begin{array}{ccc} & & \left|\;\int_{L_{2}^{\nu }\left[0,T\right]}\;(\;i\;\sum_{j=1}^{\nu }\;\left[\;I,v_{j}\left(t\right),w_{j}\left(t\right)\;\right]\;)\;\exp\{\;i\sum_{j=1}^{\nu }\;\left[\;I,v_{j}\left(t\right),x_{j}\left(t\right)\;\right]\;\}df\left(\vec{v}\right)\;\right|\\ & \leq & \int_{L_{2}^{\nu }\left[0,T\right]}\;|\;\sum_{j=1}^{\nu }\int_{0}^{T}\;v_{j}\left(t\right)\;w_{j}^{{}^{\prime }}\left(t\right)\;dt\;|\;d\left|f\right|\left(\vec{v}\right)\\ & \leq & \int_{L_{2}^{\nu }\left[0,T\right]}\;\;\sum_{j=1}^{\nu }\;||v_{j}{\left|\right|}_{2}\;\cdot \;||w_{j}^{{}^{\prime }}{\left|\right|}_{2}\;\;\;d\left|f\right|\left(\vec{v}\right)\\ & \leq & \int_{L_{2}^{\nu }\left[0,T\right]}\;\;\sum_{j=1}^{\nu }\;||v_{j}{\left|\right|}_{2}\;\cdot \;[\;{Max}_{1\leq j\leq \nu }\left|\right|w_{j}^{{}^{\prime }}{\left|\right|}_{2}\;]\;\;d|f|\left(\vec{v}\right)\\ & = & M\;\cdot \;\int_{L_{2}^{\nu }\left[0,T\right]}\;\;\sum_{j=1}^{\nu }\;||v_{j}{\left|\right|}_{2}\;\;d\left|f\right|\left(\vec{v}\right)\\ & < & \infty \;, \end{array}$$

where $M\;=\;{Max}_{1\leq j\leq \nu }\left|\right|w_{j}^{{}^{\prime }}{\left|\right|}_{2}<\infty$.  ☐

**Theorem** **5.** (34) $$\begin{array}{cc} (1). & \;E_{\vec{x}}^{anw_{z}}(D\left[F,\vec{x}+\vec{y},\vec{w}\;\right])\\ = & \int_{L_{2}^{\nu }\left[0,T\right]}(\;i\sum_{j=1}^{\nu }\left[I,v_{j}\left(t\right),w_{j}\left(t\right)\right]\;)\;\exp\{-\frac{1}{2z}\sum_{j=1}^{\nu }\int_{0}^{T}v_{j}^{2}\left(s\right)ds+i\sum_{j=1}^{\nu }\left[I,v_{j}\left(t\right),y_{j}\left(t\right)\right]\}df\left(\vec{v}\right). \end{array}$$

(35) $$\begin{array}{cc} (2). & \;E_{\vec{x}}^{anf_{q}}(D\left[F,\;\vec{x}+\vec{y},\;\vec{w}\right]\;)\;)\\ = & \int_{L_{2}^{\nu }\left[0,T\right]}(\;i\;\sum_{j=1}^{\nu }\;\left[\;I,v_{j}\left(t\right),w_{j}\left(t\right)\;\right]\;)\;\exp\{-\frac{i}{2q}\sum_{j=1}^{\nu }\int_{0}^{T}v_{j}^{2}\left(s\right)ds+i\sum_{j=1}^{\nu }\;\left[\;I,v_{j}\left(t\right),y_{j}\left(t\right)\;\right]\;\}df\left(\vec{v}\right). \end{array}$$

**Proof** **.** (1). For $z\in C_{+}$,

(36) $$\begin{array}{ccc} & & E_{\vec{x}}^{anw_{z}}(D\left[F,\vec{x}+\vec{y},\;\vec{w}\;\right])\\ & = & E_{\vec{x}}(D\left[F,z^{-\frac{1}{2}}\vec{x}+\vec{y},\;\vec{w}\;\right])\\ & = & E_{\vec{x}}(\int_{L_{2}^{\nu }\left[0,T\right]}\;(\;i\;\sum_{j=1}^{\nu }\;\;\left[\;I,v_{j}\left(t\right),w_{j}\left(t\right)\;\right]\;)\\ & & \;\;\;\cdot \;\exp\{iz^{-\frac{1}{2}}\;\;\left[\;I,v_{j}\left(t\right),x_{j}\left(t\right)\;\right]\;+\;i\;\sum_{j=1}^{\nu }\;\;\left[\;I,v_{j}\left(t\right),y_{j}\left(t\right)\;\right]\;\}\;df\left(\vec{v}\right)\;)\\ & = & \int_{L_{2}^{\nu }\left[0,T\right]}\;(\;i\;\sum_{j=1}^{\nu }\;\left[\;I,v_{j}\left(t\right),w_{j}\left(t\right)\;\right]\;)\cdot \;[E_{\vec{x}}(\exp\{iz^{-\frac{1}{2}}\sum_{j=1}^{\nu }\;\left[\;I,v_{j}\left(t\right),x_{j}\left(t\right)\;\right]\;\})\\ & & \;\;\;\cdot \;\exp\{i\;\sum_{j=1}^{\nu }\;\left[\;I,v_{j}\left(t\right),y_{j}\left(t\right)\;\right]\;\}\;df\left(\vec{v}\right)\\ & = & \int_{L_{2}^{\nu }\left[0,T\right]}\;(\;i\;\sum_{j=1}^{\nu }\;\left[I,v_{j}\left(t\right),w_{j}\left(t\right)\right]\;)\cdot \;\exp\{-\frac{1}{2z}\;\sum_{j=1}^{\nu }\left|\right|v_{j}{\left|\right|}_{2}^{2}\;\}\\ & & \;\;\;\cdot \;\exp\{i\;\sum_{j=1}^{\nu }\;\left[\;I,v_{j}\left(t\right),y_{j}\left(t\right)\;\right]\;\}\;df\left(\vec{v}\right)\\ & = & \int_{L_{2}^{\nu }\left[0,T\right]}(\;i\sum_{j=1}^{\nu }\left[I,v_{j}\left(t\right),w_{j}\left(t\right)\;\right])\;\exp\{-\frac{1}{2z}\sum_{j=1}^{\nu }\left|\right|v_{j}{\left|\right|}_{2}^{2}+i\sum_{j=1}^{\nu }\left[I,v_{j}\left(t\right),y_{j}\left(t\right)\right]\}df\left(\vec{v}\right) \end{array}$$

(2).

(37) $$\begin{array}{ccc} & & E_{\vec{x}}^{anf_{q}}(D\left[F,\;\vec{x}+\vec{y},\;\vec{w}\right]\;)\;)\\ & = & \lim_{z\to -iq}E_{\vec{x}}^{anw_{z}}(D\left[F,\vec{x}+\vec{y},\;\vec{w}\;\right])\\ & = & \lim_{z\to -iq}\int_{L_{2}^{\nu }\left[0,T\right]}(i\;\sum_{j=1}^{\nu }\;\left[I,v_{j}\left(t\right),w_{j}\left(t\right)\right])\cdot \exp\{-\frac{1}{2z}\sum_{j=1}^{\nu }\left|\right|v_{j}{\left|\right|}_{2}^{2}+i\;\sum_{j=1}^{\nu }\left[I,v_{j}\left(t\right),y_{j}\left(t\right)\right]\}df\left(\vec{v}\right)\\ & = & \int_{L_{2}^{\nu }\left[0,T\right]}(i\sum_{j=1}^{\nu }\left[I,v_{j}\left(t\right),w_{j}\left(t\right)\right])\cdot \exp\{-\frac{i}{2q}\sum_{j=1}^{\nu }\int_{0}^{T}v_{j}^{2}\left(s\right)ds+i\sum_{j=1}^{\nu }\left[I,v_{j}\left(t\right),y_{j}\left(t\right)\right]\}df\left(\vec{v}\right) \end{array}$$

 ☐

**Lemma** **5.** *For $\lambda \in C_{+}$,*

(38) $$\begin{array}{ccc} & & \exp\{\frac{1-\lambda }{2}\;\sum_{j=1}^{\nu }\sum_{k=1}^{n}\;{\left[I,\phi_{k}\left(t\right),x_{j}\left(t\right)\;\right]}^{2}\;\}\;D\left[F,\vec{x}+\vec{y},\vec{w}\;\right]\; \end{array}$$

*is a Wiener-integrable function of $\vec{x}\in C_{0}^{\nu }\left[0,T\right]$.*

**Proof.**  First we have

(39) $$\begin{array}{ccc} & & E_{\vec{x}}(\exp\{\frac{1-\lambda }{2}\sum_{j=1}^{\nu }\sum_{k=1}^{n}\;{\left[\;I,\phi_{k}\left(t\right),x_{j}\left(t\right)\;\right]}^{2}\}D\left[F,\vec{x}+\vec{y},\vec{w}\;\right])\\ & = & E_{\vec{x}}(\exp\{\frac{1-\lambda }{2}\sum_{k=1}^{n}\;(\sum_{j=1}^{\nu }{\left[I,\phi_{k}\left(t\right),x_{j}\left(t\right)\;\right]}^{2}\}\\ & & \;\cdot \;[\int_{L_{2}^{\nu }\left[0,T\right]}\;(\;i\;\sum_{j=1}^{\nu }\;\left[I,\;v_{j}\left(t\right)\;,w_{j}\left(t\right)\;\right]\;)\\ & & \;\cdot \;\exp\{i\;\sum_{j=1}^{\nu }\;\left[I,v_{j}\left(t\right),x\left(t\right)\;\right]\;+\;i\;\sum_{j=1}^{\nu }\;\left[I,v_{j}\left(t\right),y_{j}\left(t\right)\;\right]\;\}\;df\left(\vec{v}\right))\\ & = & \int_{L_{2}^{\nu }\left[0,T\right]}\;(\;i\;\sum_{j=1}^{\nu }\left[\;I,v_{j}\left(t\right)\;,w_{j}\left(t\right)\;\right]\;)\cdot \;E_{\vec{x}}(\exp\{\frac{1-\lambda }{2}\sum_{j=1}^{\nu }\sum_{k=1}^{n}\;{\left[I,\phi_{k}\left(t\right),x_{j}\left(t\right)\;\right]}^{2}\}\\ & & \;\cdot \;\exp\{i\;\sum_{j=1}^{\nu }\;\left[I,v_{j}\left(t\right),x_{j}\left(t\right)\;\right]\;+\;i\;\sum_{j=1}^{\nu }\;\left[I,v_{j}\left(t\right),y_{j}\left(t\right)\right]\})df\left(\vec{v}\right)\;. \end{array}$$

Therefore we have

(40) $$\begin{array}{ccc} & & \left|E_{\vec{x}}(\exp\{\frac{1-\lambda }{2}\sum_{j=1}^{\nu }\sum_{k=1}^{n}\;{\left[\;I,\phi_{k}\left(t\right),x_{j}\left(t\right)\;\right]}^{2}\}D\left[F,\vec{x}+\vec{y},\vec{w}\;\right])\;\right|\\ & \leq & \int_{L_{2}^{\nu }\left[0,T\right]}\;|\;\sum_{j=1}^{\nu }\;\left[I,\;v_{j}\left(t\right),\;w_{j}\left(t\right)\;\right]\;|\cdot \;E_{\vec{x}}(\;|\exp\{\frac{1-\lambda }{2}\sum_{j=1}^{\nu }\sum_{k=1}^{n}\;{\left[I,\phi_{k}\left(t\right),x_{j}\left(t\right)\right]}^{2}\}\;|\;)d\left|f\right|\left(\vec{v}\right)\\ & \leq & \int_{L_{2}^{\nu }\left[0,T\right]}|\;\sum_{j=1}^{\nu }\;\left[I,\;v_{j}\left(t\right),\;w_{j}\left(t\right)\;\right]\;|\;\cdot \;|\;{\left(2\pi \right)}^{-\frac{n}{2}}\int_{R^{\nu n}}\;\exp\{-\frac{\lambda }{2}\sum_{j=1}^{\nu }\sum_{k=1}^{n}\;u_{j,k}^{2}\}\;d\vec{u}\;|\;d\left|f\right|\left(\vec{v}\right)\\ & = & \int_{L_{2}\left[0,T\right]}\;|\;\sum_{j=1}^{\nu }\;\left[I,\;v_{j}\left(t\right),\;w_{j}\left(t\right)\;\right]\;\;|\;\cdot \;|{\left(2\pi \right)}^{-\frac{\nu n}{2}}{\left(\frac{2\pi }{\lambda }\right)}^{\frac{\nu n}{2}}|\;d\left|f\right|\left(\vec{v}\right)\\ & = & \lambda^{-\frac{\nu n}{2}}\;\;\;\int_{L_{2}^{\nu }\left[0,T\right]}\;|\;\sum_{j=1}^{\nu }\;\left[I,\;v_{j}\left(t\right),\;w_{j}\left(t\right)\;\right]\;|\;d\left|f\right|\left(\vec{v}\right)\\ & < & \infty \;, \end{array}$$

using Lemma 4.  ☐

**Theorem** **6.** *For $z\in C_{+}$,*

(41) $$\begin{array}{ccc} & & E_{\vec{x}}^{anw_{z}}(D\left[F,\vec{x}+\vec{y},\vec{w}\;\right])\\ & = & \lim_{n\to \infty }z^{\frac{\nu n}{2}}\cdot E_{\vec{x}}(\exp\{\frac{z-1}{2\;z}\sum_{j=1}^{\nu }\sum_{k=1}^{n}{\left[I,\phi_{k}\left(t\right),x_{j}\left(t\right)\;\right]}^{2}\}D\left[F,\vec{x}+\vec{y},\vec{w}\right])\;. \end{array}$$

**Proof** **.** **By Lemma 4,**

(42) $$\begin{array}{ccc} & & D[F,z^{-\frac{1}{2}}\vec{x}+\vec{y},\vec{w}\;]\;\\ & = & \int_{L_{2}^{\nu }\left[0,T\right]}(\;i\sum_{j=1}^{\nu }\left[I,v_{j}\left(t\right),w_{j}\left(t\right)\right]\;)\\ & & \;\;\;\exp\{\sum_{j=1}^{\nu }(iz^{-\frac{1}{2}}\left[I,v_{j}\left(t\right),x_{j}\left(t\right)\right]+i\left[I,v_{j}\left(t\right),y_{j}\left(t\right)\right])\}df\left(\vec{v}\right)\;\;\; \end{array}$$

By Lemma 3,

(43) $$\begin{array}{ccc} & & \lim_{n\to \infty }\;z^{\frac{\nu n}{2}}\;\cdot \;E_{\vec{x}}(\exp\{\frac{z-1}{2\;z}\sum_{j=1}^{\nu }\sum_{k=1}^{n}{\left[I,\phi_{k}\left(t\right),x_{j}\left(t\right)\;\right]}^{2}\}D\left[F,\vec{x}+\vec{y},\vec{w}\right])\\ & = & \lim_{n\to \infty }z^{\frac{\nu n}{2}}\;\int_{L_{2}^{\nu }\left[0,T\right]}E_{\vec{x}}(\exp\{\frac{z-1}{2\;z}\sum_{j=1}^{\nu }\sum_{k=1}^{n}{\left[I,\phi_{k}\left(t\right),x_{j}\left(t\right)\;\right]}^{2}\}\;\\ & & \;\;\;\;\cdot \;\exp\{i\;\sum_{j=1}^{\nu }\left[I,v_{j}\left(t\right),x_{j}\left(t\right)\right]\}\;\;)\\ & & \;\;\;\;\cdot \;(\;i\;\sum_{j=1}^{\nu }\left[\;I,v_{j}\left(t\right),w_{j}\left(t\right)\;\right]\;)\cdot \;\exp\{i\;\sum_{j=1}^{\nu }\left[\;I,v_{j}\left(t\right),y_{j}\left(t\right)\;\right]\}df\left(\vec{v}\right)\\ & = & \lim_{n\to \infty }z^{\frac{\nu n}{2}}\;\int_{L_{2}^{\nu }\left[0,T\right]}\;z^{-\frac{\nu n}{2}}\;\cdot \;\exp\{\frac{z-1}{2\;z}\sum_{j=1}^{\nu }\sum_{k=1}^{m}{\left[\int_{0}^{T}\;\phi_{k}\left(s\right)\;v_{j}\left(s\right)\;ds\;\right]}^{2}\;-\;\frac{1}{2}\sum_{j=1}^{\nu }\int_{0}^{T}v_{j}^{2}\left(t\right)dt\}\\ & & \;\;\;\;\cdot \;(\;i\;\sum_{j=1}^{\nu }\left[\;I,v_{j}\left(t\right),w_{j}\left(t\right)\;\right]\;)\cdot \;\;\exp\{i\;\sum_{j=1}^{\nu }\left[\;I,v_{j}\left(t\right),y_{j}\left(t\right)\;\right]\}df\left(\vec{v}\right)\\ & = & \lim_{n\to \infty }\;\int_{L_{2}^{\nu }\left[0,T\right]}\;(\;i\;\sum_{j=1}^{\nu }\left[I,\;v_{j}\left(t\right),w_{j}\left(t\right)\right]\;)\cdot \;\;\exp\{-\frac{1}{2\;z}\sum_{j=1}^{\nu }\sum_{k=1}^{n}{\left[\int_{0}^{T}\;\phi_{k}\left(s\right)\;v_{j}\left(s\right)\;ds\;\right]}^{2}\;\}\;\\ & & \;\;\;\;\cdot \;\exp\{\frac{1}{2}\sum_{j=1}^{\nu }\sum_{k=1}^{n}\;{\left[\;\int_{0}^{T}\;\phi_{k}\left(s\right)\;v_{j}\left(s\right)\;ds\;\right]}^{2}\;-\;\frac{1}{2}\sum_{j=1}^{\nu }\int_{0}^{T}\;v_{j}^{2}\left(t\right)\;dt\}\\ & & \;\;\;\;\cdot \;\;\exp\{i\;\sum_{j=1}^{\nu }\left[\;I,v_{j}\left(t\right),y_{j}\left(t\right)\;\right]\}df\left(\vec{v}\right)\\ & = & \int_{L_{2}^{\nu }\left[0,T\right]}\;(i\;\sum_{j=1}^{\nu }\left[\;I,v_{j}\left(t\right),w_{j}\left(t\right)\;\right])\cdot \;\exp\{-\frac{1}{2z}\;\sum_{j=1}^{\nu }\left|\right|v_{j}{\left|\right|}_{2}^{2}\;+\;i\;\sum_{j=1}^{\nu }\left[\;I,v_{j}\left(t\right),y_{j}\left(t\right)\;\right]\}df\left(\vec{v}\right)\\ & = & \int_{L_{2}^{\nu }\left[0,T\right]}\;(i\;\sum_{j=1}^{\nu }\left[\;I,v_{j}\left(t\right),w_{j}\left(t\right)\;\right])\cdot \;E_{\vec{x}}(\exp\{iz^{-\frac{1}{2}}\sum_{j=1}^{\nu }\left[\;I,v_{j}\left(t\right),x_{j}\left(t\right)\;\right]\})\\ & & \;\;\;\;\;\cdot \;\exp\{i\;\sum_{j=1}^{\nu }\left[\;I,v_{j}\left(t\right),y_{j}\left(t\right)\;\right]\}df\left(\vec{v}\right)\\ & = & E_{\vec{x}}(\int_{L_{2}^{\nu }\left[0,T\right]}\;(i\;\sum_{j=1}^{\nu }\left[\;I,v_{j}\left(t\right),w_{j}\left(t\right)\;\right])\\ & & \;\;\;\;\cdot \;\exp\{iz^{-\frac{1}{2}}\sum_{j=1}^{\nu }\left[\;I,v_{j}\left(t\right),x_{j}\left(t\right)\;\right]\;+\;i\;\sum_{j=1}^{\nu }\left[\;I,v_{j}\left(t\right),y_{j}\left(t\right)\;\right]\}df\left(\vec{v}\right))\\ & = & E_{\vec{x}}(D\left[F,z^{-\frac{1}{2}}\vec{x}+\vec{y},\vec{w}\;\right])\\ & = & E_{\vec{x}}^{anw_{z}}(D\left[F,\vec{x}+\vec{y},\vec{w}\;\right])\;. \end{array}$$

 ☐

**Theorem** **7.** *For real ρ>0,*

(44) $$\begin{array}{ccc} & & E_{\vec{x}}(D\left[F,\rho \;\vec{x}+\vec{y},\vec{w}\;\right]\;)\\ & = & \lim_{n\to \infty }\rho^{-\nu n}\;\cdot \;E_{\vec{x}}(\exp\{\frac{\rho^{2}-1}{2\rho^{2}}\sum_{j=1}^{\nu }\sum_{k=1}^{m}{\left[I,\phi_{k}\left(t\right),x_{j}\left(t\right)\;\right]}^{2}\}D\left[F,\vec{x}+\vec{y},\vec{w}\right]\;)\;. \end{array}$$

**Proof.**  For real λ>0,

(45) $$\begin{array}{ccc} & & E_{\vec{x}}^{anw_{\lambda }}(D\left[F,\vec{x}+\vec{y},\vec{w}\;\right])\\ & = & E_{\vec{x}}(D\left[F,\lambda^{-\frac{1}{2}}\vec{x}+\vec{y},\vec{w}\;\right]\;)\\ & = & \lim_{n\to \infty }\lambda^{\frac{\nu n}{2}}\;\cdot \;E_{\vec{x}}(\exp\{\frac{\lambda -1}{2\lambda }\sum_{j=1}^{\nu }\sum_{k=1}^{m}\;{\left[I,\phi_{k}\left(t\right),x_{j}\left(t\right)\;\right]}^{2}\}D\left[F,\vec{x}+\vec{y},\vec{w}\right]\;)\;. \end{array}$$

Taking $\lambda =\rho^{-2}$, Equation (44) holds.  ☐

**Theorem** **8.** (46) $$\begin{array}{ccc} & & E_{\vec{x}}^{anf_{q}}(D\left[F,\vec{x}+\vec{y},\vec{w}\;\right]\;))\\ & = & \lim_{n\to \infty }{\lambda_{n}}^{\frac{\nu n}{2}}\;\cdot \;E_{\vec{x}}(\exp\{\frac{\lambda_{n}-1}{2\lambda_{n}}\sum_{j=1}^{\nu }\sum_{k=1}^{m}{\left[I,\phi_{k}\left(t\right),x_{j}\left(t\right)\;\right]}^{2}\}D\left[F,\vec{x}+\vec{y},\vec{w}\right]\;) \end{array}$$

*whenever $\{\lambda_{n}\}\;\to -\;iq$ through $C_{+}$.*

**Proof.**  (47) $$\begin{array}{ccc} & & E_{\vec{x}}^{anf_{q}}(D\left[F,\vec{x}+\vec{y},\vec{w}\;\right]\;))\\ & = & \lim_{n\to \infty }E_{\vec{x}}^{anw_{\lambda_{n}}}(D\left[F,\vec{x}+\vec{y},\vec{w}\right]\;)\\ & = & \lim_{n\to \infty }{\lambda_{n}}^{\frac{\nu n}{2}}\;\cdot \;E_{\vec{x}}(\exp\{\frac{\lambda_{n}-1}{2\lambda_{n}}\sum_{j=1}^{\nu }\sum_{k=1}^{m}{\left[I,\phi_{k}\left(t\right),x_{j}\left(t\right)\;\right]}^{2}\}D\left[F,\vec{x}+\vec{y},\vec{w}\right]\;)\; \end{array}$$

whenever $\{\lambda_{n}\}\to -\;iq$ through $C_{+}$.  ☐

## 5. Conclusions

We prove very harmonious relationships among the integral transform and function space integrals exploiting the partial derivative on the function space.

**Remark** **2.** *In this paper, we prove new theorems by extending those results in [11,19] to the first variation theory in [1] and to the Integral Transform in [5].*

**Remark** **3.** *The author presented this paper in the conference, “The First International Workshop: Constructive Mathematical Analysis” in Selcuk University, Konya, Turkey (2019). Title, abstract and references were introduced in the proceeding (http://constructivemathematicalanalysis.com).*

## References

[1] Cameron R.H. (1951). The first variation of an indefinite Wiener integral. Proc. Am. Soc..

[2] Cameron R.H. (1954). The translation pathology of Wiener space. Duke Math. J..

[3] Cameron R.H., Martin W.T. (1944). On transformations of Wiener integrals under translations. Ann. Math..

[4] Cameron R.H., Martin W.T. (1945). Transformations for Wiener integrals under a general class of linear transformations. Trans. Am. Math. Soc..

[5] Cameron R.H., Storvick D.A. (1976). An *L*_2_-analytic Fourier Feynman transforms. Mich. Math. J..

[6] Huffman T., Park C., Skoug D. (1995). Analytic Fourier Feynman transforms and convolution. Trans. Am. Math. Soc..

[7] Huffman T., Park C., Skoug D. (1996). Convolution and Fourier Feynman transforms of functions involving multiple integrals. Mich. Math. J..

[8] Huffman T., Park C., Skoug D. (1997). Convolution and Fourier Feynman transforms. Rocky Mt. J. Math..

[9] Johnson G.W., Skoug D.L. (1979). An *L*_*p*_-analytic Fourier Feynman transforms. Mich. Math. J..

[10] Cameron R.H., Martin W.T. (1947). The behavior of measure and measurability under change of scale in Wiener space. Bull. Am. Math. Soc..

[11] Cameron R.H., Storvick D.A. (1987). Change of scale formulas for Wiener integral. Suppl. Rendicoti Circ. Mat. Palermo Ser. II—Numero.

[12] Cameron R.H., Storvick D.A. (1988). Relationships between the Wiener integral and the analytic Feynman integral. Suppl. Rendicoti Circ. Mat. Palermo Ser. II—Numero.

[13] Kim Y.S. (2001). Relationships between Fourier Feynman transforms and Wiener integrals on abstract Wiener spaces. Integral Transform. Spec. Funct..

[14] Kim Y.S. (2018). Behavior of a scale factor for Wiener integrals and a Fourier Stieltjes transform on the Wiener space. Appl. Math..

[15] Kim Y.S. (2005). Relationships between Fourier Feynman transforms and Wiener integrals on abstract Wiener spaces II. Integral Transform. Spec. Funct..

[16] Kim Y.S. (2016). Behavior of the first variation of a measure on the Fourier Feynman Transform and Convolution. Numer. Funct. Anal. Optim..

[17] Kim Y.S. (2006). The behavior of the first variation under the Fourier Feynman transform on abstract Wiener spaces. J. Fourier Anal. Appl..

[18] Kim Y.S. (2010). Fourier Feynman Transform and analytic Feynman integrals and convolutions of a Fourier transform μ of a measure on Wiener spaces. Houst. J. Math..

[19] Cameron R.H., Storvick D.A. (1980). Some Banach algebras of analytic Feynman integrable functionals, an analytic functions. Lect. Notes Math..

