# Correction: Young Sik, K. Partial Derivative Approach to the Integral Transform for the Function Space in the Banach Algebra. *Entropy* 2020, *22*, 1047

**DOI:** 10.3390/e22121369

**Published:** 2020-12-03

**Authors:** Kim Young Sik

**Affiliations:** Department of Mathematics, Hanyang University, Seoul 04763, Korea; yoskim@hanyang.ac.kr

## 1. Correction for Equations

In the original article [1], there were some mistakes in Equations as published.

(1) We mistyped $\frac{1-z}{2}$ as $\frac{z-1}{2z}$, and $\frac{1-\lambda }{2}$ as $\frac{\lambda -1}{2\lambda }$, and $\frac{1-\lambda_{n}}{2}$ as $\frac{\lambda_{n}-1}{2\lambda_{n}}$ in Equation (23), Equation (25), Equations (27)–(29), Equation (41), Equation (43) and Equations (45)–(47). We correct them:
  (23) $$\lim_{n\to \infty }z^{\frac{n}{2}}\;\cdot \;E_{x}(\exp\{\frac{1-z}{2}\sum_{k=1}^{n}\;{\left[I,\;\phi_{k}\left(t\right)\;,x\left(t\right)\;\right]}^{2}\}\;\left[D,F,x+y,w\right]).$$
  (25) $$\begin{array}{c} \lim_{n\to \infty }\;z^{\frac{n}{2}}\;\cdot E_{x}(\exp\{\frac{1-z}{2}\sum_{k=1}^{n}{\left[I,\;\phi_{k}\left(t\right),\;x\left(t\right)\;\right]}^{2}\}\;\left[D,F,x+y,w\right])\\ =\lim_{n\to \infty }z^{\frac{n}{2}}\;\int_{L_{2}\left[0,T\right]}\;E_{x}(\exp\{\frac{1-z}{2}\sum_{k=1}^{n}{\left[I,\phi_{k}\left(t\right),x\left(t\right)\right]}^{2}\;+i\;\left[I,v\left(t\right),x\left(t\right)\right]\;\})\\ \;\;\cdot (i[I,v(t),w(t)])\cdot \exp\{i[I,v(t),y(t)]\}df(v). \end{array}$$
  (27) $$\lim_{n\to \infty }\lambda^{\frac{n}{2}}\;\cdot \;E_{x}(\exp\{\frac{1-\lambda }{2}\sum_{k=1}^{m}\;{\left[\;I\;\phi_{k}\left(t\right)\;x\left(t\right)\;\right]}^{2}\}\;\left[D,F,x+y,w\right)])\;.$$
  (28) $$\lim_{n\to \infty }{\lambda_{n}}^{\frac{n}{2}}\;\cdot \;E_{x}(\exp\{\frac{1-\lambda_{n}}{2}\sum_{k=1}^{m}{\left[I,\phi_{k}\left(t\right),x\left(t\right)\right]}^{2}\;\}\;\left[D,F,x+y,w\right)])\;.$$
  (29) $$\lim_{n\to \infty }{\lambda_{n}}^{\frac{n}{2}}\;\cdot \;E_{x}(\exp\{\frac{1-\lambda_{n}}{2}\sum_{k=1}^{m}{\left[I,\phi_{k}\left(t\right),x\left(t\right)\right]}^{2}\;\}\;\left[D,F,x+y,w\right)])\;.$$
  (41) $$\lim_{n\to \infty }z^{\frac{\nu n}{2}}\cdot E_{\vec{x}}(\exp\{\frac{1-z}{2}\sum_{j=1}^{\nu }\sum_{k=1}^{n}{\left[I,\phi_{k}\left(t\right),x_{j}\left(t\right)\;\right]}^{2}\}\left[D,F,\vec{x}+\vec{y},\vec{w}\right)])\;.$$
  (43) $$\begin{array}{c} \lim_{n\to \infty }\;z^{\frac{\nu n}{2}}\;\cdot \;E_{\vec{x}}(\exp\{\frac{1-z}{2}\sum_{j=1}^{\nu }\sum_{k=1}^{n}{\left[I,\phi_{k}\left(t\right),x_{j}\left(t\right)\;\right]}^{2}\}\left[D,F,\vec{x}+\vec{y},\vec{w}\right)]\\ =\lim_{n\to \infty }z^{\frac{\nu n}{2}}\;\int_{L_{2}^{\nu }\left[0,T\right]}E_{\vec{x}}(\exp\{\frac{1-z}{2}\sum_{j=1}^{\nu }\sum_{k=1}^{n}{\left[I,\phi_{k}\left(t\right),x_{j}\left(t\right)\;\right]}^{2}\}\\ \;\;\;\cdot \exp\{i\sum_{j=1}^{\nu }\left[I,v_{j}\left(t\right),x_{j}\left(t\right)\right]\})\\ \;\cdot (i\sum_{j=1}^{\nu }\left[I,v_{j}\left(t\right),w_{j}\left(t\right)\right])\cdot \exp\{i\sum_{j=1}^{\nu }\left[I,v_{j}\left(t\right),y_{j}\left(t\right)\right]\}df\left(\vec{v}\right). \end{array}$$
  (45) $$=\lim_{n\to \infty }\lambda^{\frac{\nu n}{2}}\;\cdot \;E_{\vec{x}}(\exp\{\frac{1-\lambda }{2}\sum_{j=1}^{\nu }\sum_{k=1}^{m}\;{\left[I,\phi_{k}\left(t\right),x_{j}\left(t\right)\;\right]}^{2}\}\left[D,F,\vec{x}+\vec{y},\vec{w}\right)]\;)\;.$$
  (46) $$=\lim_{n\to \infty }{\lambda_{n}}^{\frac{\nu n}{2}}\;\cdot \;E_{\vec{x}}(\exp\{\frac{1-\lambda_{n}}{2}\sum_{j=1}^{\nu }\sum_{k=1}^{m}{\left[I,\phi_{k}\left(t\right),x_{j}\left(t\right)\;\right]}^{2}\}\left[D,F,\vec{x}+\vec{y},\vec{w}\right)]\;).$$
  (47) $$=\lim_{n\to \infty }{\lambda_{n}}^{\frac{\nu n}{2}}\;\cdot \;E_{\vec{x}}(\exp\{\frac{1-\lambda_{n}}{2}\sum_{j=1}^{\nu }\sum_{k=1}^{m}{\left[I,\phi_{k}\left(t\right),x_{j}\left(t\right)\;\right]}^{2}\}\left[D,F,\vec{x}+\vec{y},\vec{w}\right)]\;).$$
(2) In Equations (26)–(29):
  (a). We mistyped [D,F,x+y,w] as D[F,x+y,w] in Equations (26)–(29).
  (b). We mistyped [D,F,ρx+y,w] as D[F,ρx+y,w] in Equation (26).
(3) In Equations (31)–(47):
  (a). We mistyped $\left[D,F,\vec{x},\vec{w}\right]$ as $D[F,\vec{x},\vec{w}]$ in Equations (31) and (32).
  (b). We mistyped $\left[D,F,\vec{x}+\vec{y},\vec{w}\right]$ as $D[F,\vec{x}+\vec{y},\vec{w}]$ in Equations (34)–(41) and Equations (43)–(47).
  (c). We mistyped $\left[D,F,z^{-\frac{1}{2}}\vec{x}+\vec{y},\vec{w}\right]$ as $D[F,z^{-\frac{1}{2}}\vec{x}+\vec{y},\vec{w}]$ in Equations (42) and (43).
  (d). We mistyped $\left[D,F,\rho \vec{x}+\vec{y},\vec{w}\right]$ as $D[F,\rho \vec{x}+\vec{y},\vec{w}]$ in Equation (44).
  (e). We mistyped $\left[D,F,\lambda^{-\frac{1}{2}}\vec{x}+\vec{y},\vec{w}\right]$ as $D[F,\lambda^{-\frac{1}{2}}\vec{x}+\vec{y},\vec{w}]$ in Equation (45).

The authors apologize for any inconvenience caused and state that the scientific conclusions are unaffected. The original article has been updated.
